# Cholinergic Modulation of Disorder-Relevant Neural Circuits in Generalized Anxiety Disorder

**DOI:** 10.1016/j.biopsych.2019.12.013

**Published:** 2020-05-15

**Authors:** Toby Wise, Fiona Patrick, Nicholas Meyer, Ndaba Mazibuko, Alice E. Oates, Anne H.M. van der Bijl, Philippe Danjou, Susan M. O’Connor, Elizabeth Doolin, Caroline Wooldridge, Deborah Rathjen, Christine Macare, Steven C.R. Williams, Adam Perkins, Allan H. Young

**Affiliations:** a Department of Psychological Medicine, Institute of Psychiatry, Psychology & Neuroscience, King’s College London, London, UK; b Department of Neuroimaging, Institute of Psychiatry, Psychology & Neuroscience, King’s College London, London, UK; c Department of Psychosis Studies, Institute of Psychiatry, Psychology & Neuroscience, King’s College London, London, UK; d Wellcome Trust Centre for Neuroimaging, University College London, London, UK; e Max Planck UCL Centre for Computational Psychiatry and Ageing Research, London, UK; f National Institute for Health Research Biomedical Research Centre, South London, London, UK; g Maudsley NHS Foundation Trust, London, UK; h Department of Humanities and Social Sciences, California Institute of Technology, Pasadena, California; i Faculty of Social and Behavioural Sciences, University of Leiden, Leiden, Netherlands; j Biotrial, Paris, France; k Bionomics Ltd, Thebarton, Australia; l BiOasis Technologies Inc., Guilford, Connecticut

**Keywords:** Amygdala, Anterior cingulate cortex, Cholinergic modulation, Generalized anxiety disorder, fMRI, Pharmacotherapy

## Abstract

**Background:**

Generalized anxiety disorder is associated with hyperactivity in the amygdala-prefrontal networks, and normalization of this aberrant function is thought to be critical for successful treatment. Preclinical evidence implicates cholinergic neurotransmission in the function of these systems and suggests that cholinergic modulation may have anxiolytic effects. However, the effects of cholinergic modulators on the function of anxiety-related networks in humans have not been investigated.

**Methods:**

We administered a novel α7 nicotinic acetylcholine receptor–negative allosteric modulator, BNC210, to 24 individuals (3 male subjects) with generalized anxiety disorder and assessed its effects on neural responses to fearful face stimuli.

**Results:**

BNC210 reduced amygdala reactivity to fearful faces relative to placebo and similarly to lorazepam and also reduced connectivity between the amygdala and the anterior cingulate cortex, a network involved in regulating anxious responses to aversive stimuli.

**Conclusions:**

These results demonstrate for the first time that the function of disorder-relevant neural circuits in generalized anxiety disorder can be beneficially altered through modulation of cholinergic neurotransmission and suggest potential for this system as a novel target for anxiolytic pharmacotherapy.

Generalized anxiety disorder (GAD) is both common and debilitating (1), characterized by a pathologically elevated level of anxiety that interferes with daily life. Despite this, the etiology is poorly understood, and current treatments are associated with numerous side effects, a delayed onset of therapeutic action, and low response rates (2,3). As a result, the identification of molecules with novel mechanisms of action with the potential to be more effective drug treatments is critical to improving outcomes for individuals with GAD.

Neuroimaging has demonstrated that GAD is associated with altered function within brain networks implicated in affective responses to aversive stimuli. Most notably, amygdala hyperactivity in response to threatening stimuli is a replicable finding in individuals with anxiety disorders (4,5). Anxiolytic drugs designed for both immediate and long-term use diminish this hyperreactivity in healthy control subjects (6,7) and individuals with an anxiety diagnosis (8,9), suggesting that normalization of amygdala response may be critical in treating anxiety. Amygdala activity in response to aversive stimuli is regulated through functional interactions with the anterior cingulate cortex (10,11), and functional connectivity in these circuits is elevated in clinical anxiety (12). Successful treatment of anxiety may therefore depend on normalization of function within prefrontal-amygdala networks underlying the regulation of anxious responses to threat. As such, functional magnetic resonance imaging (fMRI) explorations with novel compounds that show reduction in prefrontal-amygdala connectivity or reduced amygdala reactivity more generally could identify promising new treatments and exclude those likely to be ineffective before commencement of costly, large-scale clinical trials (13).

Although existing treatments typically target gamma-aminobutyric acidergic (GABAergic) and serotonergic neurotransmission, there is increasing evidence that cholinergic systems are important in fear-related behaviors and may play a role in clinical anxiety. Selective blocking of choline uptake has been linked to disinhibition of exploration in novel environments in rats (14), and alteration of cholinergic activity via acetylcholine inhibitor infusions has been associated with reduction of fear reactions in plus maze paradigms (15). Nicotinic acetylcholine receptors (nAChRs) appear particularly important in driving these effects; rodent studies have demonstrated increases in anxiety arising from increased nAChR activity (16), implicating nAChRs specifically in anxious behavior. In particular, α7 nAChR antagonist infusion has been linked to anxiolytic behavior (17), and the high concentration of these receptors in the amygdala suggests that the effects of nAChR modulation may be mediated by changes in amygdala function (18).

Investigation of alternative anxiolytic compounds is particularly important in the context of the limitations of currently available pharmacological interventions for anxiety. For example, physical dependence, development of tolerance, and issues with withdrawal occur when benzodiazepines are used long term (19,20), while serotonin/noradrenaline reuptake inhibitors may take weeks to develop their anxiolytic effects and involve unpleasant side effects that can impact compliance (2). However, there is no research to date investigating the role of cholinergic neurotransmission in the function of networks associated with anxiety disorders in humans, and it is unclear whether modulation of cholinergic signaling might beneficially alter pathological activity in these networks. We tested this by administering a novel α7 nAChR-negative allosteric modulator, BNC210, to individuals with GAD.

BNC210 is a selective, negative allosteric modulator of the α7 nAChR in development by Bionomics Limited (Thebarton, Australia) for the treatment of anxiety, trauma, and stressor-related disorders. It inhibits rat and human α7 nAChR currents (in stably transfected cell lines) induced by acetylcholine, nicotine, choline, and the α7-specific agonist PNU-282987, with IC50 values in the range of 1.2 to 3 μM. BNC210 does not displace alpha-bungarotoxin binding, and its inhibitory effects are not influenced by the concentration of acetylcholine used (EC20 or EC80), providing evidence that the modulation is via an allosteric site. It does not show any activity in binding, fluorescent, or electrophysiology assays at other members of the cys-loop ligand-gated ion channel family and has been screened at over 700 targets in assays ranging from binding to functional (in all modes including allosteric) (21).

In preclinical models, the anxiolytic profile of BNC210 is compelling and has been observed at low doses in several rodent species and models of anxiety (22). In addition to acute anxiolytic promise in nonclinical models of anxiety, BNC210 has demonstrated safety in human studies and lack of side effects including sedation, impaired motor coordination, cognitive impairment, development of tolerance, and physical dependence, offering several advantages over benzodiazepines and antidepressants. Effects of BNC210 on amygdala reactivity to fearful faces, a well-replicated biomarker for anxiety disorders that is known to be ameliorated by existing treatments, were assessed and validated against placebo and lorazepam (a positive control). We hypothesized that BNC210 would reduce amygdala responses to fearful faces and conducted follow-up exploratory analyses examining whether such effects were associated with altered connectivity in a wider, anxiety-related, amygdala-anterior cingulate cortex network.

## Methods and Materials

### Participants

In all, 24 volunteers with GAD participated in the study. Participants were recruited via online advertisements that contained a web link for a 50-item online version of the Trait Self-Description Inventory, a nonclinical Big Five personality questionnaire (23). Individuals scoring ≥ 1 SD than the population mean on neuroticism were invited to undergo telephone screening and if eligible were invited to a formal psychiatric and physical health screening session [further explanation of this method can be seen in Patrick *et al.* (23)]. All participants provided informed consent, and the study was approved by the London-Chelsea Research Ethics Committee.

GAD diagnosis was established in accordance with DSM-IV, using the Mini-International Neuropsychiatric Interview (24), administered by clinicians trained in the study protocol. Participants were excluded on the basis of a score >15 on the Montgomery–Åsberg Depression Rating Scale (25); any significant cardiovascular, gastrointestinal, hepatic, renal, respiratory, endocrine, immunologic, or hematological disease; use of any prescription or over-the-counter drug within less than 5 times the elimination half-life preceding dosing (with the exception of contraceptive medications); and use of any prescription drug that is a potential cytochrome P450 3A4 inducer within 30 days prior to dosing. Participants were all right-handed. Participants were to have refrained from smoking within the preceding 3 months and were breath-tested at each visit to ensure compliance. Participants were all currently treatment free (including psychological therapies). Participants did not hold a prescription for benzodiazepines and were drug screened at each visit.

### Experimental Procedure

**Figure 1:**
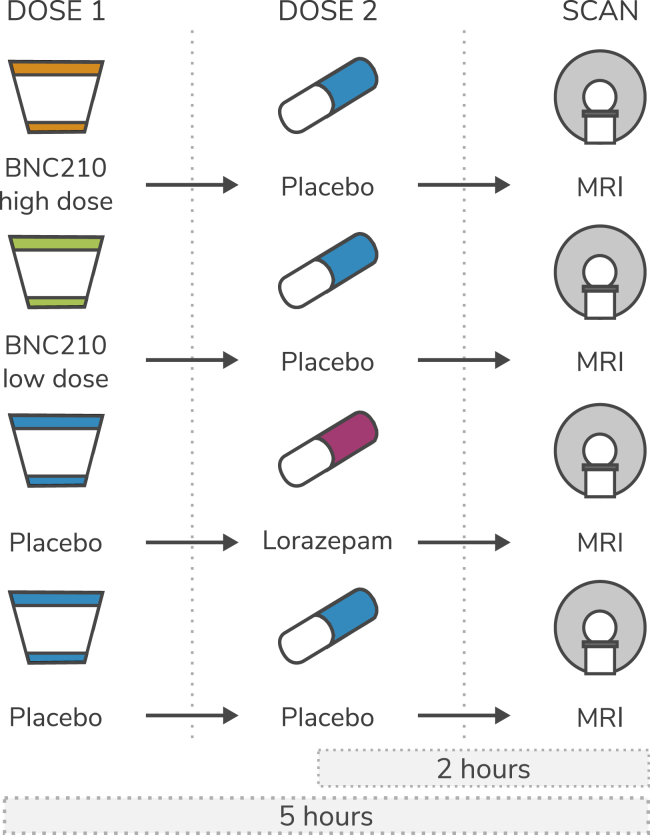
Illustration of the four dosing procedures. MRI, magnetic resonance imaging.

This study constituted a 4-way crossover, double-blind, randomized controlled trial. The novel compound, BNC210, was orally administered as a liquid suspension at 2 strengths (a low dose of 300 mg and a high dose of 2000 mg). BNC210 was previously investigated in 4 clinical trials (at single doses ranging from 5 to 2000 mg) with 84 subjects being exposed to BNC210 at 2000 mg (oral suspension, fed). The maximum tolerated dose has not been reached in animal toxicology studies or in humans, and no safety concerns have been identified at any dose. Maximum exposure from single doses has been achieved with the 2000-mg liquid suspension formulation. Based on anxiolytic activity in animal models, 300 mg is the anticipated clinically effective human dose; however, the highest dose of 2000 mg was also used to maximize the opportunity of achieving high enough exposure to detect a response in the brain. Exposure is not linear between the 300-mg and 2000-mg doses; the increase is approximately 4-fold (protocol BNC210.002 listed on http://www.anzctr.org.au). Capsule lorazepam at 1.5 mg was orally administered as the active control compound [doses of 1.5–3 mg have been recommended as anxiolytic in patient populations, with greater than 2 mg suggested as sedative or causing substantial psychomotor slowing (26,27)]. Placebo was presented as either a liquid or a capsule, depending on which medication it was replacing (Figure 1).

Following inclusion in the trial, participants attended 4 dosing sessions, receiving 1 of the 4 interventions (high-dose BNC210, low-dose BNC210, lorazepam, or placebo) at each visit in a randomized order. These were spaced with a minimum of 5 days between dosing to allow for pharmacological washout. Before each dosing, participants were given a medical health check and a standardized, high-fat breakfast. They were also instructed to avoid all caffeine-containing products for 12 hours before the visit.

As the absorption rates of BNC210 and lorazepam differ, with time taken to reach peak concentration occurring at approximately 5 hours and 2 hours, respectively, the dosing schedule incorporated two dose administrations per visit, with one always being a placebo, to maintain the blind for both participants and researchers. For the placebo condition, subjects were administered two placebos at 5 and 2 hours before testing, in line with the administration times for the active drugs.

### Symptom Measures

At enrollment, participants completed the Big Five personality questionnaire and the Hamilton Anxiety Rating Scale (HAM-A) (28). Self-report anxiety data were also recorded at 3 time points—before first drug administration, immediately before scanning, and following completion of fMRI acquisition—using the state subscale of the State-Trait Anxiety Inventory (29).

### fMRI Acquisition

fMRI was performed 5 hours after first drug/placebo administration. Data were acquired on a MR750 3-Tesla scanner with a 12-channel head coil (GE Healthcare, Chicago, IL); 180 volumes were acquired per functional run using a T2*-weighted echo-planar imaging sequence (repetition time = 2000 ms, echo time = 30 ms, field of view = 22.1 cm, flip angle = 75°, 41 slices, resolution = 3.3 mm^3^), with 4 initial volumes discarded to allow for magnetization equilibration effects. Cardiac signals were recorded with a plethysmograph, while respiration was measured using a respiratory belt. A high-resolution T1-weighted image was also acquired (repetition time = 7.31 ms, echo time = 3.02 ms, 256 × 256 matrix, 196 slices, voxel size = 1.2 × 1.05 × 1.05 mm).

### Emotional Faces Task

We used an emotional faces processing task previously shown to activate the amygdala (30,31). Faces of three emotions (fearful, happy, sad) at two intensities (medium and high) taken from the Ekman emotional faces set (32) were displayed for 2 seconds each, interleaved with neutral faces. Each emotion was shown in a separate run, each lasting approximately 6 minutes. To encourage implicit processing of the emotional faces in the task (33), participants reported the gender of the faces using a button box with their right hand. This has been shown to produce robust effects and to detect disorder-related alterations in emotion processing (34,35). Here, we focused on the fearful face condition given our hypotheses regarding threat-related processing; however, results for the other emotions are provided in Supplemental Table S2.

### fMRI Preprocessing

Data were preprocessed with custom Nipype (http://nipy.org/nipype/) scripts, using tools from SPM12 (http://www.fil.ion.ucl.ac.uk/spm) and custom code. Images were initially realigned to the first image of the first session, slice timing corrected, and co-registered to the high-resolution T1 image from the first session. Segmentation and normalization were performed on the T1 image, and deformation fields were then used to normalize the functional images to MNI space before smoothing with a 6-mm full width at half maximum kernel. Subjects exhibiting substantial translation of over 1 voxel (3 mm) were removed from further analyses to prevent contamination by gross head movement. Volumes exhibiting high levels of motion were identified using ArtifactDetect, implemented in Nipype, based on realignment parameters and volume-to-volume signal intensity changes. Physiological signals were processed using custom scripts implementing the RETROICOR algorithm (36) to produce cardiac and respiratory regressors for use in first-level analyses.

### Statistical Analysis

Data were analyzed using SPM12. First-level models were formed including regressors for each emotion at each level (neutral, medium, high), along with 6 motion parameters generated during realignment, and physiological regressors generated by the RETROICOR procedure. We also included motion “scrubbing” regressors to exclude volumes exhibiting high motion from the analysis, a procedure that has been demonstrated to increase statistical power in fMRI analysis and reduce confounding effects of motion (37).

The contrast of interest for these analyses was formed by comparing the medium- and high-intensity fear emotions against fixation (modeled as an implicit baseline condition). This approach was chosen in favor of a contrast against neutral faces owing to substantial evidence indicating that anxious individuals perceive affectively neutral expressions as negative (38), suggesting that neutral faces would not represent true affective neutrality.

We tested for drug effects using paired *t* tests against placebo in SPM12, with head motion, measured as the total distance traveled, as a covariate. In addition, *F* tests across all drug conditions are reported in Supplemental Table S2. We used a region of interest (ROI) approach with the MarsBaR toolbox (www.marsbar.sourceforge.net) to compare mean activity in our a priori amygdala ROI while viewing fearful faces between drug conditions, correcting for multiple comparisons across the two hemispheres using false discovery rate (FDR) correction. To maximize our power to detect changes in fear-relevant processing, the chosen ROI was derived from the Neurosynth (www.neurosynth.org) map for the term “fear” to identify regions most associated with responses to fear. The conjunction of this map and the Automated Anatomical Labelling atlas (39) amygdala regions was used to create ROIs. All ROI analyses were FDR corrected for multiple comparisons across the two hemispheres.

We also performed exploratory whole-brain analyses to identify other effects on reactivity to fearful faces. These analyses used a cluster-defining threshold of *p* < .001 and a clusterwise threshold of *p* < .05, FDR corrected for multiple comparisons across the whole brain.

### Functional Connectivity

To assess whether drug effects on amygdala reactivity were part of a wider response within anxiety-relevant amygdala-prefrontal networks, we performed generalized psychophysiological interaction analyses for drug conditions showing significant effects on amygdala reactivity using the generalized psychophysiological interaction toolbox (https://www.nitrc.org/projects/gppi). For each participant, two analyses were performed with the extracted first eigenvariate time courses of the left and right amygdala ROIs, using the same fear versus baseline contrast as the main analyses. Group-level effects were examined using a 2 × 2 analysis of variance with seed hemisphere and drug as factors and head motion as a covariate. To test for effects of the drug, accounting for hemispheric differences, we conducted a *t* test on the main effect of drug condition. Given a large body of evidence showing modulatory effects of the anterior cingulate cortex on amygdala activity (10) and associations between connectivity in this network and anxiety (11,12), we used an ROI approach with masks taken from the Automated Anatomical Labelling anterior and midcingulate regions. Results were thresholded with a voxelwise threshold of *p* < .001 uncorrected and a clusterwise threshold of *p* < .05, FDR corrected within the a priori selected ROI. We also report whole-brain results for completeness.

## Results

### Participants

Three subjects were removed from analyses owing to excessive motion. Table 1 shows the average age and HAM-A and neuroticism personality scores for the sample; HAM-A scores of 17 sit within the mild to moderate range of clinical anxiety (40). None of the participants met the Mini-International Neuropsychiatric Interview criteria for major depressive disorder or scored over 15 on the Montgomery–Åsberg Depression Rating Scale as per the eligibility criteria. The final sample included 21 individuals (19 female, 2 male). There was no significant difference between genders in HAM-A scores.

**Table 1:** Age at Enrollment and Hamilton Anxiety Rating Scale (HAM-A) and Neuroticism Scores at Screening

Measure	Mean	SD
Age, Years	28.4	6.5
HAM-A Score	17.8	9.7
Neuroticism Score	53.4	6.6

### Behavioral Results

Participants demonstrated high accuracy on the gender discrimination task (mean = 87.8%, SD = 4.4%). None of the drug conditions differed significantly from placebo in accuracy (lowest *p* = .14); however, reaction times were significantly slower than placebo in the lorazepam condition (*t*_20_ = 5.53, *p* < .001, *d* = 1.21), suggesting task vigilance but motor slowing in line with known effects of benzodiazepines.

### Motion Differences Between Drug Conditions

There were no differences in total distance traveled between drug conditions, although the BNC210 high-dose condition showed a trend toward a reduction in movement (lorazepam: *t*_20_ = −1.04, *p* = .31; BNC210 low dose: *t*_20_ = 0.18, *p* = .86; BNC210 high dose: *t*_20_ = −1.80, *p* = .09). Nonetheless, to remove motion-related variance, motion was included as a covariate in further analyses.

### Viewing Fearful Faces Increases Amygdala Activity

To assess the effects of the emotion manipulation in the absence of drug effects and confirm that the manipulation was activating the amygdala as expected, we explored the effects of viewing fearful faces in the placebo condition. As expected, results revealed a significant response to fearful faces in both left (*t*_20_ = 5.32, *p* < .001) and right (*t*_20_ = 4.51, *p* < .001) amygdala. Whole-brain analysis revealed several other regions responding to fearful faces, which are described in Supplemental Table S1. There were no significant correlations between amygdala reactivity to fearful faces and trait anxiety, state anxiety before scanning, or neuroticism.

### BNC210 Reduces Amygdala Reactivity to Fearful Faces

**Figure 2:**
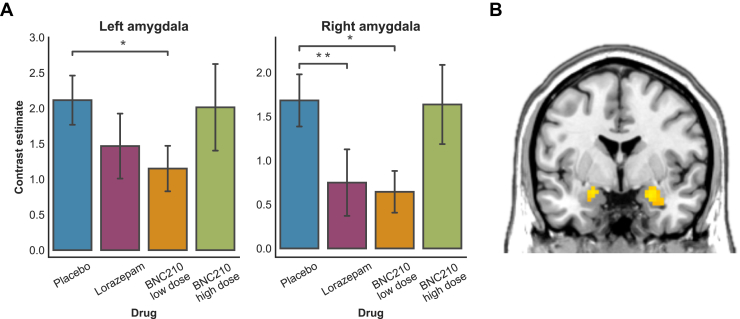
**(A)** Mean (±SE) amygdala response to fearful faces for each drug condition. **(B)** Effect of BNC210 low dose on amygdala activity, thresholded at *p* < .05 uncorrected for illustration. *p < .05, **p < .01.

Relative to placebo, the low dose of BNC210 significantly reduced left (*t*_20_ = 2.78, *p* = .011) and right (*t*_20_ = 3.07, *p* = .006) amygdala reactivity (Figure 2A, B). Lorazepam significantly reduced activity in the right (*t*_19_ = 2.21, *p* = .047) but not left (*t*_19_ = 1.78, *p* = .09) amygdala. There was no difference between placebo and BNC210 high dose (*p* = .33, both left and right amygdala). Whole-brain exploratory analyses showed no effect of either drug in other regions on activity while viewing fearful faces.

### BNC210 Decreases Amygdala-Anterior Cingulate Functional Connectivity

**Figure 3:**
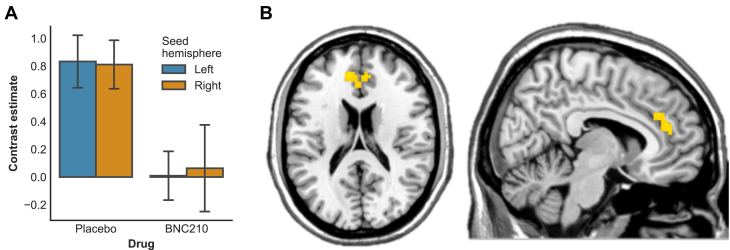
**(A)** Mean functional connectivity strength with amygdala seeds while viewing fearful faces in the anterior cingulate cortex cluster. **(B)** Anterior cingulate cortex cluster showing significant reductions in functional connectivity strength with the amygdala under BNC210 (low dose) in the psychophysiological interaction analysis.

To investigate whether reductions in amygdala reactivity were associated with reduced connectivity in a larger anxiety-relevant network, we performed generalized psychophysiological interaction analyses on connectivity between the amygdala and anterior cingulate. This revealed a reduction in connectivity between the amygdala and anterior cingulate cortex while viewing fearful faces under the BNC210 low dose (peak = −12, 41, 14; *t* = 4.22; *p* = .012, FDR corrected) (Figure 3A, B). This remained significant at the whole-brain level (peak = −9, 41, 20; *t* = 4.24; *p* = .018, FDR corrected); however, no other significant clusters emerged. There were no differences in connectivity between placebo and lorazepam conditions, and connectivity strength in the placebo condition did not correlate significantly with state anxiety before scanning or neuroticism.

## Discussion

We investigated the effects of a novel α7 nAChR-negative allosteric modulator, BNC210, on anxiety-relevant neural circuits in individuals with GAD using fMRI. Our results demonstrate that a low dose of BNC210 reduces amygdala responses to fearful faces and reduces task-related anterior-cingulate functional connectivity in a sample of individuals with GAD, suggesting for the first time that cholinergic neurotransmission plays a critical role in the function of these networks and that drugs targeting this system may be beneficial in the treatment of anxiety disorders.

Amygdala hyperactivity to threat-related stimuli is a well-described marker of anxiety (4,5), and drugs targeting both GABAergic and serotonergic systems have been shown to reduce amygdala activity to these stimuli (6,41), suggesting that normalization of amygdala response may be critical for the therapeutic effects of anxiolytic medication. This effect has also been shown in response to no pharmacological frontline interventions, such as cognitive behavioral therapy (42), suggesting that interventions leading to reduction in amygdala hyperactivity hold promise. Our results indicate that negative allosteric modulation of α7 nAChR by BNC210 reduces amygdala reactivity in line with approved therapeutic interventions through its actions on cholinergic neurotransmission.

We also showed that BNC210 reduces amygdala-anterior cingulate functional connectivity, which is elevated in clinical anxiety (12) and is implicated in anxious responses to aversive stimuli (11,43). Connectivity within this network is affected by manipulations of serotonergic neurotransmission (44), suggesting that normalization of hyperactivity in this network may mediate the actions of serotonin-targeting pharmacotherapy on symptoms of anxiety. Our results demonstrate that negative allosteric modulation of the α7 nAChR receptor, and hence reduced action of acetylcholine at synapses expressing these receptors, reduces connectivity between the amygdala and anterior cingulate cortex. This indicates that cholinergic neurotransmission plays an important role in the functioning of this network and provides more evidence for the potential of cholinergic modulation in modulating anxiety-relevant neurocircuitry.

Lorazepam significantly reduced right but not left amygdala reactivity to fearful faces. It should be noted that the relatively modest effects of lorazepam compared with previous work may result from the dose of the drug used here. We purposely selected a moderate dose to prevent unblinding by the sedative effects of lorazepam at higher doses, and this may have diminished its effects at the neural level. The half-life of lorazepam is around 60 to 120 minutes (6); as our task was administered at 120 minutes from ingestion of lorazepam, this may have also had an effect.

The effects of BNC210 may be explained by suppressive action on glutamatergic interneurons in the basolateral amygdala. The amygdala is subject to both excitatory and inhibitory regulation from glutamatergic and GABAergic interneurons, respectively, and the action of these interneurons is dependent on cholinergic activity at α7 receptors (45). Although preclinical evidence suggests that α7 stimulation increases inhibitory activity in interneurons of the amygdala, potentially through upregulation of GABAergic inhibitory interneurons (45) resulting in reduced responses to direct stimulation, clinical anxiety may be associated with relatively increased excitatory glutamatergic interneuron activity, in which case BNC210’s effects could be explained by α7-mediated downregulation of these excitatory inputs to the amygdala, in line with previous animal work (16).

This study has several strengths. First, the double-blind, double-dummy crossover design reduced expectation effects and minimized between-subject variability in responses. Additionally, the use of lorazepam as a positive control treatment enabled us to ensure that negative results were not due to an insensitivity of the design to pharmacological manipulations of emotion processing, as lorazepam has previously been shown to reduce amygdala reactivity to threat. A further strength of this work was the use of physiological and motion-related denoising methods in the fMRI preprocessing and analysis to maximize power and to reduce influence of confounding factors.

The absence of a healthy control group is a limitation of this study, although if BNC210 is indeed normalizing anxiety-related hyperresponsive neurocircuitry, it is possible that no effect would have been seen in healthy individuals. Although patients were excluded if their Montgomery–Åsberg Depression Rating Scale score was >15, subjects did exhibit some symptoms of depression as expected owing to the high level of comorbidity between these conditions; however, none of the participants reached a clinically diagnostic level, and depressive symptoms are thus not expected to affect findings. Indeed, the presence of symptoms related to depression suggests that the present sample is in line with common presentations of GAD. Length of GAD symptomatic experience (beyond the 6 months specified by the Mini-International Neuropsychiatric Interview) was not recorded, and in accordance with the requirements of the trial, participants were currently treatment free; however, all the participants met diagnostic criteria for GAD as determined by structured interview and experienced psychiatrists. There is some evidence suggesting that anxiolytics may have altered effect depending on menstrual cycle stage (46), although this is not unanimously agreed (47); no information regarding this was collected, so potential effects cannot be commented on here.

Finally, we found no relationship between amygdala reactivity or amygdala-anterior cingulate connectivity with self-report measures of anxiety or neuroticism. However, as all participants in the study had a highly anxious personality profile, this part of the analysis is affected by range restriction, especially as state anxiety scores are typically highly positively correlated with trait anxiety scores (e.g., *r* = .63) (48); hence, maintaining the high state anxiety score is, to a degree, recapitulating the participants’ personality (trait) profile. Future work could explore the use of in-scanner visual analog scales to achieve a more nuanced measure of subjective anxiety with closer temporal proximity to task.

This study provides a basis for future work on cholinergic systems in anxiety and raises several interesting questions. Firstly, the exact mechanisms through which modulation of cholinergic neurotransmission leads to changes in amygdala reactivity remain unclear, although existing preclinical work suggests directions for future work in this area. Secondly, it is not clear how cholinergic modulation affects amygdala-anterior cingulate connectivity, particularly whether this is due to top-down effects of BNC210 in the anterior cingulate cortex or to bottom-up effects at the amygdala that drive anterior cingulate activity. This is a challenging question to address in human fMRI, which animal work could help to answer. It is also of interest that only the low dose of BNC210 had a significant effect on amygdala reactivity, and this raises the possibility of the dose-response relationship being U-shaped rather than linear. The specificity of BNC210 for the α7 nAChR has been explored in prior studies (21,22), identifying no “off-target” effects that might otherwise explain these data. This supports the suggestion that the findings may represent homeostatic rebalancing; α7 nAChR is expressed on both GABAergic and glutamatergic interneurons in the amygdala (and hippocampus) (45), indicating that it may have a role in maintaining the inhibitory/excitatory balance in these locations. Finally, it remains to be seen whether cholinergic modulation through agents such as BNC210 is effective in treating symptoms of clinical anxiety, and future studies will investigate this.

In conclusion, our study is the first to demonstrate that modulation of cholinergic neurotransmission has a normalizing effect on exaggerated threat-related amygdala function in GAD, suggesting a new target for pharmacological treatment for anxiety disorders.

## Supplementary Material

Supplemental Tables S1 and S2

Key Resources Table
