# Supplementary material for: Cholinergic Modulation of Disorder-Relevant Neural Circuits in Generalized Anxiety Disorder
Source: Biol Psychiatry. 2020 May 15;87(10):908–15. doi: 10.1016/j.biopsych.2019.12.013 (PMC7198974; doi:10.1016/j.biopsych.2019.12.013)
Supplement: Supplemental Tables S1 and S2 [file mmc1.pdf]

Cholinergic Modulation of Disorder-Relevant Neural Circuits in Generalized Anxiety Disorder

Supplemental Information

Table S1. Activation elicited by viewing fearful faces in the placebo condition (*p* < .05 FDR corrected). There were no clusters that showed significant deactivation in the fearful face condition relative to baseline.

Region	Brodmann areas	Cluster extent (voxels)	*T*	*p*	MNI coordinates
Cerebellum, lingual gyrus, superior occipital gyrus, middle occipital gyrus, calcarine sulcus	18, 19	7730	10.56	< .001	12, -55, -19
Precentral gyrus	6	118	6.63	< .001	45, -7, 53
Inferior frontal gyrus	44	166	6.41	< .001	42, 17, 20
Inferior frontal gyrus	44	44	6.13	0.007	-33, 14, 23
Middle frontal gyrus	46	91	5.87	< .001	-27, 41, 26
Superior frontal gyrus	46	73	5.8	0.001	24, 44, 14
Postcentral gyrus, angular gyrus	40, 7	201	5.61	< . 001	27, -37, 44
Supramarginal gyrus	42	45	4.89	0.007	60, -40, 26

Table S2. Comparisons between drug conditions for all emotions. F-tests represent the overall main effect of drug in an ANOVA, while t-tests represent the comparison between each drug and placebo. Degrees of freedom differ across drug conditions due to differing numbers of excluded subjects based on motion. Positive *t* values represent a reduction in activity relative to placebo.

Emotion	Hemisphere	F-test	Lorazepam	BNC210 low dose	BNC210 high dose
Fear	Left	*F*(3, 69) = 1.53<br>p = 0.38	*t*(19) = 1.78<br>p = 0.088	***t*(19) = 2.78**<br>**p = 0.011**	*t*(19) = 0.2<br>p = 0.61
	Right	*F*(3, 69) = 2.56<br>p = 0.12	***t*(19) = 2.12**<br>**p = 0.047**	***t*(19) = 3.07**<br>**p = 0.006**	*t*(19) = 0.07<br>p = 0.69
Happy	Left	*F*(3, 73) = 0.62<br>p = 0.85	*t*(18) = 1.82<br>p = 0.08	*t*(18) = 0.97<br>p = 0.31	*t*(18) = 0.97<br>p = 0.31
	Right	*F*(3, 73) = 0.19<br>p = 0.99	*t*(18) = 0.80<br>p = 0.39	*t*(18) = 1.30<br>p = 0.20	*t*(18) = 0.46<br>p = 0.54
Sad	Left	*F*(3, 61) = 2.01<br>p = 0.22	***t*(15) = 2.74**<br>**p = 0.015**	*t*(15) = 1.03<br>p = 0.30	*t*(15) = -0.98<br>p = 0.97
	Right	*F*(3, 61) = 0.81<br>p = 0.74	*t*(15) = 1.64<br>p = 0.12	*t*(15) = 0.58<br>p = 0.49	*t*(15) = -0.31<br>p = 0.85
